# Adverse events and dose modifications of tyrosine kinase inhibitors in chronic myelogenous leukemia

**DOI:** 10.3389/fonc.2022.1021662

**Published:** 2022-10-06

**Authors:** Kota Yoshifuji, Koji Sasaki

**Affiliations:** ^1^ Department of Hematology, Tokyo Medical and Dental University, Tokyo, Japan; ^2^ Department of Leukemia, The University of Texas MD Anderson Cancer Center, Houston, TX, United States

**Keywords:** chronic myelogenous leukemia, imatinib, dasatinib, nilotinib, bosutinib, ponatinib, asciminib, dose modification

## Abstract

The prognosis of chronic myelogenous leukemia (CML-CP) in chronic phase has improved dramatically since the introduction of imatinib. In addition to imatinib, second- and third-generation tyrosine kinase inhibitors (TKIs) and a novel allosteric inhibitor, asciminib, are now available. During long-term TKI therapy, the optimal selection of TKI therapy for individual patients requires the understanding of specific patterns of toxicity profile to minimize chronic toxicity and the risk of adverse events, including pulmonary arterial hypertension, pleural effusion, and cardiovascular events. Given the high efficacy of TKI therapy, dose modifications of TKI therapy reduce the risk of toxicities and improves quality of life during therapy. In this review article, we summarize the characteristics and adverse event profile of each TKI and dose modifications in patients with CML-CP and discuss future perspectives in the treatment of CML-CP.

## Introduction

Chronic myelogenous leukemia (CML) is a clonal myeloproliferative neoplasm derived from pluripotent hematopoietic stem cells (1) and characterized by the presence of Philadelphia chromosome, which is caused by the reciprocal translocation of the *ABL1* (Abelson murine leukemia) gene on the long arm of chromosome 9 and the *BCR* (breakpoint cluster region) gene on the long arm of chromosome 22. The BCR::ABL1 protein constitutively activates downstream signaling pathways such as the Ras, phosphatidylinositol-3 kinase (PI3K)/Akt, and JAK/STAT pathways, which induce tumor growth (2). The incidence of CML varies between 10 and 15 cases/10^6^ people/year without significant regional or racial differences (3); the median age at diagnosis ranges from 55 to 65 years (3–5).

The development of imatinib, a BCR::ABL1 tyrosine kinase inhibitor (TKI), dramatically improved the prognosis of CML (6, 7). Subsequently, second-generation TKIs dasatinib, nilotinib, and bosutinib and third-generation TKI, ponatinib, which shows efficacy in refractory cases with T315I mutation, have been approved by the Food and Drug Administration in the United States. More recently, asciminib, a first-in-class TKI that inhibits BCR::ABL1 by binding to the ABL myristoyl pocket, has been approved for CML treatment (8). These TKI therapy has achieved favorable survival in patients with CML similar to that of general population, particularly in patients who achieved remission (9–15). Therefore, the optimal selection of TKI, the management of TKI toxicity, and the monitoring of response are the key to improve quality of life during long-term TKI therapy and to prevent TKI-related complications given the near-normal life expectancy in patients with CML (16).

Treatment-free remission (TFR) is now an emerging therapeutic goal for patients in sustained deep molecular response (DMR) (17–19). Approximately, half of the patients maintained DMR without TKI therapy (20–22). However, a long-term TKI therapy is required before discontinuation. Therefore, we must remain vigilant for adverse events associated with TKI therapy.

In addition to BCR::ABL1, each TKI has its specific activity against various other kinases (off-target effect) (23). The variation of the inhibition on other kinases lead to a wide range of symptomatic toxicities: Imatinib may cause fluid retention and muscle cramps (24, 25); dasatinib, pulmonary arterial hypertension and pleural effusion (26, 27); nilotinib, hyperglycemia, hyperlipidemia, pancreatitis, and cardiovascular events (28, 29); bosutinib, hepatic impairment and diarrhea (30); and ponatinib, hypertension, pancreatitis, and arterial occlusive events (31). Although the exact cause of dasatinib-related pleural effusion is unknown, it has been hypothesized that this adverse event may be immune-mediated based on reports of elevated lymphocyte counts in pleural fluid and tissue (32). Dasatinib-related pleural effusion may also result from platelet derived growth factor receptor (PDGFR) β inhibition, which lowers interstitial fluid pressure, or from SRC-family kinases inhibition, which alters vascular permeability (33). Several mechanisms have been reported for the pathogenesis of arterial occlusive events. Nilotinib and ponatinib inhibit c-KIT and PDGFR, affecting vascular endothelial cells and perivascular cells, delaying vascular injury recovery and inhibiting angiogenesis (34–36). Nilotinib inhibits discoidin domain receptor 1 (DDR1), which is involved in plague formation, and promotes atherosclerosis (37). Ponatinib causes hypertension *via* inhibition of the vascular endothelial growth factor receptor (VEGFR) (38). Moreover, in a recent report, in which pro-inflammatory cytokines (IL6 and TNFα) and anti-inflammatory cytokine (IL10) of patients with CML were prospectively measured, nilotinib, not dasatinib and imatinib, induced an imbalance between pro- and anti- inflammatory cytokines. This imbalance led to an excess of inflammation and might help to promote atherothrombotic events (39). To reduce these adverse events, appropriate TKI selection is necessary in light of the patient’s underlying comorbidities. Moreover, the significance of TKI dose modification is also attractive attention (40).

In this review, we first describe the characteristics and adverse event profile of each TKI approved for CML treatment, and then we discuss the latest findings on TKI dose modification in patients with CML to reduce adverse events.

## Characteristics and adverse event profiles of tyrosine kinase inhibitors

### Imatinib

Imatinib, the first TKI, inhibits the tyrosine kinase by targeting the ATP-binding site of BCR::ABL1. Imatinib also inhibits PDGFR and KIT kinase activities. The phase 3 IRIS trial demonstrated that first-line treatment of CML with imatinib, at a dose of 400 mg once daily, was more effective and led to fewer adverse events than combination treatment with interferon alfa and cytarabine (24). The 18-month cumulative complete cytogenetic response (CCyR) rates were 76.2% and 14.5% in the imatinib and the combination of interferon alfa and cytarabine, respectively (P<0.001). A long-term follow-up study of patients in the IRIS trial demonstrated favorable outcome with a 10-year survival of 83.3% in the imatinib group (25).

The STIM study, the first prospective multicenter study of imatinib discontinuation in patients with CML who maintained DMR for at least 2 years, revealed that 41% of patients maintained DMR at 12 months (20). Subsequently, the A-STIM study reported the fluctuations of BCR::ABL1 transcript levels below the major molecular response (MMR) threshold and concluded the loss of MMR is a practical threshold to resume TKI therapy. In the A-STIM study, 64% of patients maintained MMR at 12 months after imatinib discontinuation (41).

Imatinib may affect quality of life through chronic common adverse events, including edema, nausea, muscle spasms, and rash (24). However, serious adverse events were uncommon during the first 12 months of treatment (25). Cardiovascular events have been reported in 7.1% of patients treated with imatinib, and second neoplasms (benign or malignant) have been observed in 11.3% (25).

### Dasatinib

Dasatinib, a second-generation TKI, has 325 times the potency of imatinib against unmutated BCR::ABL1 *in vitro* (42). Dasatinib also inhibits Src family kinases, as off-target effects. The open-label, multinational, randomized phase 3 DASISION trail revealed that dasatinib 100 mg/day induced significantly higher and faster CCyR and MMR than imatinib 400 mg/day. The 12-month CCyR and MMR rate was 77% and 46% in the dasatinib group and 66% and 28% in the imatinib group, respectively (26).

Several trials of dasatinib discontinuation have been conducted in patients with CML (21, 43–45). The DADI study evaluated the treatment-free remission in patients who received dasatinib as a first-line treatment, and reported that 55.2% of patients maintained DMR at least 1 year since the discontinuation of dasatinib (21).

Dasatinib-related adverse events include pleural effusion, pulmonary arterial hypertension, and bleeding diathesis. In a 5-year follow-up of the DASISION trial, the incidence of pleural effusion was 28% in the dasatinib group (27). The onset of pleural effusion was the highest in the first year with reduced incidence of subsequent years of dasatinib therapy. Patients 65 years and older were more likely to develop pleural effusions than younger patients. In retrospective real-life analysis, 196 out of 852 CML patients (23%) who were treated with dasatinib in 21 Italian hematological centers suffered from pleural effusions, and recurrence occurred in 59.4% of cases after dose reduction of dasatinib (46). In older cases, 15.4 to 28.8% of older patients who received dasatinib suffered from them (47, 48).

The first case of dasatinib-related pulmonary arterial hypertension was reported in 2009 (49). Subsequently, several cases of dasatinib-related pulmonary arterial hypertension were reported in the French pulmonary hypertension registry (50). In the 5-year follow-up of DASISION, the incidence of pulmonary arterial hypertension was 5% after years of therapy in the dasatinib group (27). In many cases, pulmonary arterial hypertension occurred several years after initiation of dasatinib, and particular attention should be paid to patients receiving long-term dasatinib treatment. Dasatinib-related pulmonary arterial hypertension typically appears to be curable if the drug is stopped (51), but sometimes irreversible cases were reported (52).

### Nilotinib

Nilotinib, a second-generation TKI, has greater potency and selectivity for BCR::ABL1 than imatinib (53). The open-label, multicenter, phase 3, randomized ENESTnd trial demonstrated that nilotinib (600 mg/day or 800 mg/day) was more efficacious than imatinib 400 mg/day. The 12-month cumulative CCyR rates were 80%, 78%, and 65% in the nilotinib 600 mg/day, nilotinib 800 mg/day and imatinib 400 mg/day, respectively (P<0.001); the 12-month cumulative MMR rates were 44%, 43%, and 22% in the nilotinib 600 mg/day, nilotinib 800 mg/day, and imatinib 400 mg/day, respectively (P<0.001) (28).

Several prospective nilotinib discontinuation trials have been conducted (54–56). The ENESTfreedom study, a TKI discontinuation study for patients who received front-line nilotinib for more than 2 years and achieved DMR for at least 1 year, reported that 51.6% of patients maintained MMR at 48 weeks (54).

In a 5-year follow-up of the ENESTnd trial, the rates of ischemic cerebrovascular events, peripheral artery disease, and other cardiovascular events were higher in patients treated with nilotinib than in patients treated with imatinib. A positive linear relationship was observed between the cumulative incidence of cardiovascular events and treatment duration (29). Moreover, in this study, cardiovascular events were more common in patients with higher baseline Framingham general cardiovascular risk scores (57). Therefore, baseline Framingham score was predictive of cardiovascular events during nilotinib therapy (29).

### Bosutinib

Bosutinib, a second-generation TKI that targets Src and ABL, showed greater activity against BCR::ABL1 *in vitro* compared with imatinib, with minimal activity against c-KIT or PDGFR (58, 59). The open-label, multinational, phase 3, randomized BELA trial compared bosutinib (500 mg once daily) with imatinib (400 mg once daily) for patients with newly diagnosed CML; the primary endpoint of 12-omonth CCyR was not achieved (bosutinib 70% versus imatinib 68%) (60). The phase 3 BFORE trial compared bosutinib (400 mg once daily) with imatinib (400 mg once daily) for patients with newly diagnosed CML. The 12-month CCyR rates were 77.2% and 66.4% in the bosutinib and imatinib group, respectively; the 12-month MMR rates were 47.2% and 36.9%, respectively (30). The randomized trials confirmed the efficacy of frontline bosutinib at the dose of 400 mg/day compared to standard-dose imatinib.

Gastrointestinal and liver dysfunction and rash were the common adverse events during bosutinib therapy in the 5-year follow-up of BFORE (61). Diarrhea (79.9%), nausea (75%), ALT elevation (33.6%), AST elevation (25.7%), and rash (23.1%) were reported in the bosutinib group; in the bosutinib group, cardiovascular, cerebrovascular, and peripheral vascular adverse events rates were 4.9%, 0.7%, and 2.2%, respectively. Especially, diarrhea is sometimes debilitating. In BFORE trial, grade 3/4 diarrhea rates were 9.0% and 1.1% in the bosutinib and imatinib group, respectively. If diarrhea developed, patients should use antidiarrheal agents, avoid high-fat foods and alcohol, and drink sufficient fluids to avoid dehydration.

### Ponatinib

Ponatinib, the only third-generation TKI, inhibits mutated BCR::ABL1, including the T315I mutation, which confers resistance to first- and second-generation TKIs. Ponatinib also has activity against VEGFR, SRC, FGFR, and PDGFR (62). Ponatinib is approved for patients with CML who had resistance to or intolerant of previous TKI therapy. The phase 2 PACE trial demonstrated the efficacy of ponatinib at a starting dose of 45 mg once daily in patients with CML who experienced resistance to or could not tolerate dasatinib or nilotinib or had the T315I mutation (31). In a 5-year follow-up of the PACE study, 60% and 40% of the patients achieved major cytogenetic response and MMR, respectively. Moreover, 5-year progression-free and overall survival rates were 53% and 73%, respectively (31).

The prevention of arterial occlusive events, including cardiovascular, cerebrovascular, and peripheral vascular events, are the clinical key during ponatinib therapy through the optimal management of cardiovascular risk factors. In the 5-year follow-up of the PACE study, the cumulative incidence of arterial occlusive events was 31% before the recognition of the optimal prevention during ponatinib therapy. Common adverse events include rash, abdominal pain, thrombocytopenia, headache, dry skin, and constipation (31).

### Asciminib

Asciminib is a novel and selective allosteric BCR::ABL1 inhibitor that binds to the ABL myristoyl pocket (63). Asciminib has been approved for patients with CML refractory to or intolerant of previous TKI therapy. The randomized, open-label, multicenter, phase 3 ASCEMBL trial demonstrated the superior efficacy of asciminib (40 mg twice daily) to bosutinib (500 mg once daily) in patients with CML who had been treated with at least two prior TKIs (8). The MMR rates at week 24 were 25.5% and 13.2% in the asciminib and bosutinib group, respectively.

In the ASCEMBL trial, the treatment discontinuation rate was lower in the asciminib group than in the bosutinib group (5.8% versus 21.1%) (8). The incidence of hematological toxicities was higher in the asciminib group than in the bosutinib group; thrombocytopenia was the most common adverse event leading to treatment discontinuation with asciminib. The arterial occlusive event rates were 3.2% and 1.3% in the asciminib and bosutinib group, respectively.

## Dose modification of TKIs

### Imatinib

Previous studies examined the efficacy of a higher dose of imatinib (64, 65). Compared with imatinib 400 mg/day, the overall MMR rate was higher in patients receiving imatinib at the dose of 800 mg/day; grade 3 to 4 adverse events were more common in patients receiving imatinib 800 mg/day. Michel et al., in a study of Chronic Myeloid Leukemia-Study IV patients who had achieved at least a stable MMR, found that 90% of patients whose daily imatinib dose was reduced from 800 mg/day to 400 mg/day maintained MMR, with the benefit of less adverse events (66). Cervantes et al. reported that, in patients with sustained DMR to imatinib at a dose of 400 mg daily, reducing the dose to 300 mg daily significantly improved tolerability and preserved efficacy (67). Further, Russo et al. revealed that, in a study of INTERIM study patients who had received imatinib for more than 2 years and achieved MMR, stopping imatinib every 1 month allowed subjects not only to maintain MMR in 69% of patients, and no patients experienced new or more serous adverse events (68).

### Dasatinib

Dasatinib-related pleural effusion is treated *via* treatment interruption, dose reduction, diuretics, corticosteroids, or therapeutic thoracentesis. Dasatinib-related pulmonary arterial hypertension is a rare complication with possible poor prognosis when untreated (69). Therefore, the dose reduction of dasatinib may prevent the incidence of pleural effusion and pulmonary arterial hypertension. Naqvi et al. evaluated the efficacy and safety of dasatinib 50 mg/day in patients with newly diagnosed CML (70). The 6-month CCyR rate was 86%; the 12-month MMR rate was 79%. These results compared favorably with historical data from similar patients treated with dasatinib at a dose of 100 mg daily. The incidence of pleural effusion was 6%, which was significantly lower than that in the DASISION trial. Murai et al. examined very low-dose dasatinib 20 mg/day for patients older than 70 years with newly diagnosed CML (71). The 12-month MMR rate was 60%; the adverse event profile was tolerable with an incidence of pleural effusion at 8%. Other strategy to prevent dasatinib-related adverse events is “on/off” treatment. Rosee et al. retrospectively investigated on/off dasatinib regimen (3 to 5 days on, 2 to 4 days off) for CML patients with imatinib intolerance or resistance. This regimen significantly reduced adverse events such as pleural effusion and hematologic toxicity, and 58% achieved disease control (72). Moreover, therapeutic drug monitoring (TDM) is also a strategy to prevent adverse events. In the OPTIM-dasatinib study, CML patients considered overdosed after initiation of dasatinib were randomized between dose-reduction arm and standard dose arm, and dose-reduction reduced the incidence of pleural effusions (73). Now, Phase II trial evaluating TDM of dasatinib in older CML patients is ongoing (CML 12 study).

### Nilotinib

The ENESTnd study compared the efficacy and safety of nilotinib at 600 mg/day or 800 mg/day and imatinib 400 mg/day. In a 10-year follow-up of the ENESTnd study, the cardiovascular event rates were 16.5%, 23.5%, and 3.6% in the nilotinib 600 mg/day, nilotinib 800 mg/day, and imatinib 400 mg/day, respectively (74). Therefore, nilotinib 300 mg twice daily is recommended as the initial dose of nilotinib for patients with newly diagnosed CML. Rea et al. examined reducing the nilotinib dose after achieving MMR (75). In the NILO-RED study, nilotinib was reduced to only once daily after patients achieved MMR during nilotinib therapy; only two of 81 patients lost MMR after the dose reduction from twice daily to once daily; these two patients re-achieved MMR while continuing the same once-daily dose of nilotinib without TKI switch or dose escalation. Therefore, the dose reduction of nilotinib from twice daily to once daily can be considered in patients with CML after achieving MMR, though there have been no obvious reports of nilotinib dose reduction reducing the risk of cardiovascular events. In the second-line setting, 20 CML-CP patients intolerant to imatinib or dasatinib switched to nilotinib 300mg twice daily in the ENESTswift study, and 74% resolved non-hematological adverse events within 12 weeks (76).

### Bosutinib

The results of BELA and BFORE trials suggest bosutinib 400 mg daily is the optimal dose of frontline bosutinib therapy (30, 60). Though the BFORE trial had more patients 65 years or older, the treatment discontinuation rates were 22% and 29% in the BFORE and BELA trials. Therefore, the tolerability of bosutinib therapy at the lower dose led to higher rates of CCyR and MMR. Latagliata et al. retrospectively evaluated 101 patients older than 65 years who received bosutinib after resistant to or intolerant of previous TKI therapies. The starting dose of bosutinib was 500 mg/day in 25% of the patients, 400 mg/day in 7%, 300 mg/day in 33%, 200 mg/day in 24%, and 100 mg/day in 2%. Sixty seven percent of patients achieved MMR or deeper with a discontinuation rate of 26.4%. Therefore, lower dose of bosutinib can be considered in frail patients with severe comorbidities who failed multiple TKI therapies (77).

### Ponatinib

Second- and third-generation TKI therapy has increased the risk of cardiovascular events during TKI therapy. The dose reduction of potent third-generation TKI, ponatinib, can reduce the cardiovascular risk while maintaining its efficacy. Dorer et al. studied the effect of ponatinib dose intensity on arterial occlusive events using pooled data from three clinical trials of ponatinib. Ponatinib dose intensity was a strong independent predictor of increased risk of arterial occlusive events; each dose reduction by 15 mg/day reduced the risk of arterial occlusive events by 33% (78). The phase 2 OPTIC trial evaluated the efficacy and safety of ponatinib dose reduction in patients with CML-CP who had resistance to or intolerance of at least two prior TKI therapy or had T315I mutation. Patients were randomly assigned to three starting-dose ponatinib groups: ponatinib 45 mg/day, 30 mg/day, and 15 mg/day (79). The ponatinib dose was mandatory reduced to 15 mg when BCR::ABL1 transcript levels of 1% or lower on the International Scale (IS). The cumulative rates of BCR::ABL1 1% or lower (IS) were 44.1%, 29%, and 23.1% in the 45 mg/day, 30 mg/day and 15 mg/day, respectively. The arterial occlusive events of any grade were observed in 9.6%, 5.3%, and 3.2% in the 45 mg/day, 30 mg/day, and 15 mg/day, respectively. The consideration of risk/benefit balance is required to decide on the dose of ponatinib; the low-dose ponatinib 15 mg/day should be considered once patients achieved BCR::ABL1 of 1% or lower (IS). Several real-life analysis data also evaluated low-dose ponatinib regimens or full-dose induction followed by dose reduction in patients intolerant to prior TKIs, and showed its effectiveness (80–84).

## Conclusion and future perspectives

TKI therapy has normalized survival in patients with CML-CP similar to that of general population, particularly once patients achieved CCyR or deeper. Given the optimal survival during long-term TKI therapy, the monitoring of response, the prevention of adverse events, and the treatment-free remission is the clinical key in practice. Long-term follow-up of multiple randomized clinical trials clarified specific toxicities of each TKI therapy (**Table 1**). Therefore, the optimal selection of TKI therapy required the consideration of patient’s medical history and comorbidities for long-term TKI therapy.

**Table 1:** Adverse event profiles of tyrosine kinase inhibitors.

	Imatinib	Dasatinib	Nilotinib	Bosutinib	Ponatinib	Asciminib
Hematological						
Neutropenia	++	++	+	+	++	+
Thrombocytopenia	+	++	+	++	++	++
Anemia	+	+	+	+	++	+
Nonhematological						
Edema	+++	–	–	+	–	+
Nausea	++	+	+	+++	+	+
Vomiting	++	+	+	++	+	+
Muscle spasms	+++	–	–	–	–	–
Rash	+	–	+++	+	+++	–
Pleural effusion	–	+++	–	+	–	–
Headache	–	–	+	+	+++	+
Diarrhea	+	+	–	+++	+	–
Fatigue	+	+	+	+	++	+
Liver dysfunction	+	++	+++	+++	+	–
Arterial occlusive events	**-**	**+**	**++**	**-**	**+++**	**?**

The frequency of all grades of each hematological and nonhematological adverse events for various TKIs is compared. The frequency is shown in the order of +++, ++, +, -. The meaning of ? is unknown because no data has been shown about the frequency of arterial occlusive events by asciminib in the long term. These data are based on O’Brien SG, et al. N Engl J Med. 2003; 348 (11): 994-1004, Kantarjian H, et al. N Engl J Med. 2010; 362 (24): 2260-2270, Saglio G, et al. N Engl J Med. 2010; 362 (24): 2251-2259, Cortes JE, et al. J Clin Oncol. 2018; 36 (3): 231-237, Cortes JE, et al. Blood. 2018; 132 (4): 393-404, Rea D, et al. Blood. 2021; 138 (21): 2031-2041.

Dose reduction of TKI therapy after achieving remission can be considered to minimize the risk of adverse event and to improve quality of life during therapy. Dose reduction can be considered in the frontline therapy, particularly in low-risk patient and older patients. Optimal treatment strategies will be developed for each TKI to minimize the incidence and severity of adverse events. Treatment-free remission is now standard-of-care in patients who maintained DMR over years (22). However, TKI discontinuation is currently limited to a part of patients who have achieved deep molecular response. A novel strategy is required for the optimal selection of TKI therapies during long-term therapy (85). After achieving treatment-free remission, monitoring of BCR::ABL1 transcript levels is still requiredto detect loss of MMR early and restart TKI therapy even with novel therapies (86–91). Eltrombopag can be considered in managing TKI-related thrombocytopenia during TKI therapy (92).

Finally, the prevention and monitoring of anticipated TKI-related adverse events are required after the initiation of TKI therapy. The detection and pre-emptive therapy for cardiovascular risk factors prevents arterial occlusive events during TKI therapy (93). Though the relation of second neoplasm to TKI therapy or the underlying features of CML, the age-appropriate cancer screening should be considered given the 5-year cumulative incidence rate of second neoplasms at 4.4% (94–96).

## Author contributions

KY performed a literature search and wrote the initial draft. KS revised and amended the draft. All authors contributed to the article and approved the submitted version.

## Funding

This study is supported in part by the MD Anderson Cancer Center Leukemia SPORE CA100632, the Cancer Center Support Grant (CCSG) P30CA016672, and the Charif Souki Cancer Research Grant.

## Conflict of interest

KS reported honoraria from Otsuka; research funding and advisory boards from Novartis; advisory boards from Pfizer, outside the submitted work.

The remaining author declares that the research was conducted in the absence of any commercial or financial relationships that could be construed as a potential conflict of interest.

## Publisher’s note

All claims expressed in this article are solely those of the authors and do not necessarily represent those of their affiliated organizations, or those of the publisher, the editors and the reviewers. Any product that may be evaluated in this article, or claim that may be made by its manufacturer, is not guaranteed or endorsed by the publisher.
